# Non-invasive estimation of beat-by-beat aortic blood pressures from electrical impedance tomography data processed by machine learning

**DOI:** 10.1007/s10877-025-01274-2

**Published:** 2025-03-25

**Authors:** Fabian Müller-Graf, Jacob P. Thönes, Lisa Krukewitt, Paul Frenkel, Henryk Richter, Sascha Spors, Volker Kühn, Amelie R. Zitzmann, Stephan H. Boehm, Daniel A. Reuter

**Affiliations:** 1 Department of Anesthesiology, Intensive Care Medicine and Pain Therapy, University Medical Center Rostock, Schillingallee 35, 18057 Rostock, Germany; 2 Rudolf-Zenker-Institute for Experimental Surgery, University Medical Center Rostock, Schillingallee 69a, 18057 Rostock, Germany; 3 Institute of Communications Engineering, University of Rostock, Albert-Einstein-Straße 26, 18059 Rostock, Germany

**Keywords:** Hemodynamic monitoring, Blood pressure, Electrical impedance tomography, Machine learning, Arterial and aortic blood pressure

## Abstract

Hypotension in perioperative and intensive care settings is a significant risk factor associated with complications such as myocardial infarction and kidney injury thereby increasing perioperative complications and mortality. Continuous blood pressure monitoring is essential, yet challenging due to the invasive nature of current methods. Non-invasive techniques like Electrical Impedance Tomography (EIT) have been explored but face challenges in accurate and consistent blood pressure estimation. A machine learning (ML) approach was used to predict aortic blood pressures from EIT voltage measurements in landrace pigs. A convolutional neural network (CNN) was trained on a dataset of 75 298 heartbeats, to predict systolic (SAP), mean (MAP), and diastolic arterial pressures (DAP) of individuals whose arterial pressures were unknown to the algorithm. The Intraclass Correlation Coefficient (3,1) with absolute agreement (ICC) was calculated and the concordance was estimated, comparing reference blood pressure measurements and ML-derived estimates. A risk classification was estimated for the calculated blood pressure as suggested by Saugel et al. 2018. The ML-model demonstrated moderate correlations with invasive blood pressure measurements (ICC for SAP of 0.530, for MAP of 0.563, and for DAP of 0.521.) with a low risk score for 75.8% of the SAP and 64.2% of MAP estimated blood pressures. ML-techniques using EIT-voltages showed promising preliminary results in non-invasive aortic blood pressure estimation. Despite limitations in the amount of available training data and the experimental setup, this study illustrates the potential of integrating ML in EIT signal processing for real-time, non-invasive blood pressure monitoring.

## Purpose

Arterial blood pressure is the most important hemodynamic variable in the operating room and in the intensive care unit serving as the basis for hemodynamic assessment and guidance of therapy [6, 25]. Numerous expert statements have pointed out that this variable needs to be provided exactly, continuously, automatically and operator-independently, and in the best case, completely non-invasively [18]. Particularly in critically ill patients with complex hemodynamic deteriorations it is of relevance whether the central aortic pressure signal, or a peripheral, i.e. radial arterial signal is monitored: The aortic blood pressure signal is by far less affected by downstream pressure reflections, which has relevant impact when the pulse contour is to be analyzed to derive pulse pressure, pulse pressure variations, or stroke volume [11]. Additionally, intra-arterial blood pressure measurement is commonly utilized in the intensive care setting to continuously monitor critically ill patients. So far, there is no existing monitoring technology, which sufficiently fulfills all the aforementioned criteria.

Electrical impedance tomography (EIT) is a radiation-free functional imaging modality, which has been developed over the past 30 years [7]. EIT works by attaching an array of electrodes around the thorax, applying a high frequency electrical current in a pair of them and measuring the voltages between remaining electrodes. It estimates relative impedance changes within the thorax, which occur due to changes in the thoracic tissues and are most prominently linked to the air content of both lungs [7]. Therefore, EIT has gained increasing clinical impact in particular for monitoring the regional distribution of ventilation in the perioperative and intensive care setting [7].

Since changes in thoracic blood volume also induce characteristic signals in thoracic EIT applications it appeared promising to use this non-invasive technique also to asses [20] hemodynamic signals, such as arterial blood pressure [26], blood flow, and – in combination with ventilation – functional parameters of heart–lung interaction, such as pulse pressure variations or stroke volume variations [12]. Validating EIT parameters is challenging since their determination involves a number of processing steps such as the execution of EIT measurements, the generation of EIT images by image reconstruction algorithms, the definition of regions of interest, and finally the calculation of the EIT-derived parameter, which is then validated against appropriate clinical measures [3, 9]. Although EIT-based studies of hemodynamics have shown promising preliminary results, they struggled to achieve sufficient levels of generalizability to enter the clinical setting. To address this shortcoming, we introduced a novel machine learning (ML) approach to evaluate experimental EIT data and to predict aortic pressures. ML, known for its capacity to tackle non-linear and ill-posed problems, appears particularly well-suited for such applications.

By incorporating ML algorithms it was our goal to refine the extraction of clinically relevant information directly from EIT raw data without image reconstruction. In this study we employed ML techniques to predict aortic blood pressures from EIT voltage measurements in pigs.

## Methods

### Animal model and anaesthesia

EIT data from nine German Landrace pigs (12–16 weeks of age, male and female) were analyzed. Data were acquired during the course of an earlier project on drug-induced pulmonary hypertension [15–17]. These studies were approved by the governmental ethical board for animal research (Landesamt für Landwirtschaft, Lebensmittelsicherheit und Fischerei, Mecklenburg-Vorpommern, Germany; No: 7221.3–1–037/19) and were carried out in accordance with the EU-directive 2010/63/EU and the Animal Research: Reporting of In Vivo Experiments guidelines 2.0 (ARRIVE 2.0) [2]. Detailed information about the animal model, anesthesia, preparation and catheterization can be found in the above-mentioned previous publication [17]. Animals were raised by local swine farmers and transferred to the housing facilities a week prior to the experiments for acclimatizing. They were given free access to standard laboratory chow and water. Environmental enrichment in the housing of our pigs was implemented through the provision of interactive toys to stimulate play behavior and intermittent playing of a radio to facilitate acclimation to human interactions.

### Instrumentation

**Fig. 1:**
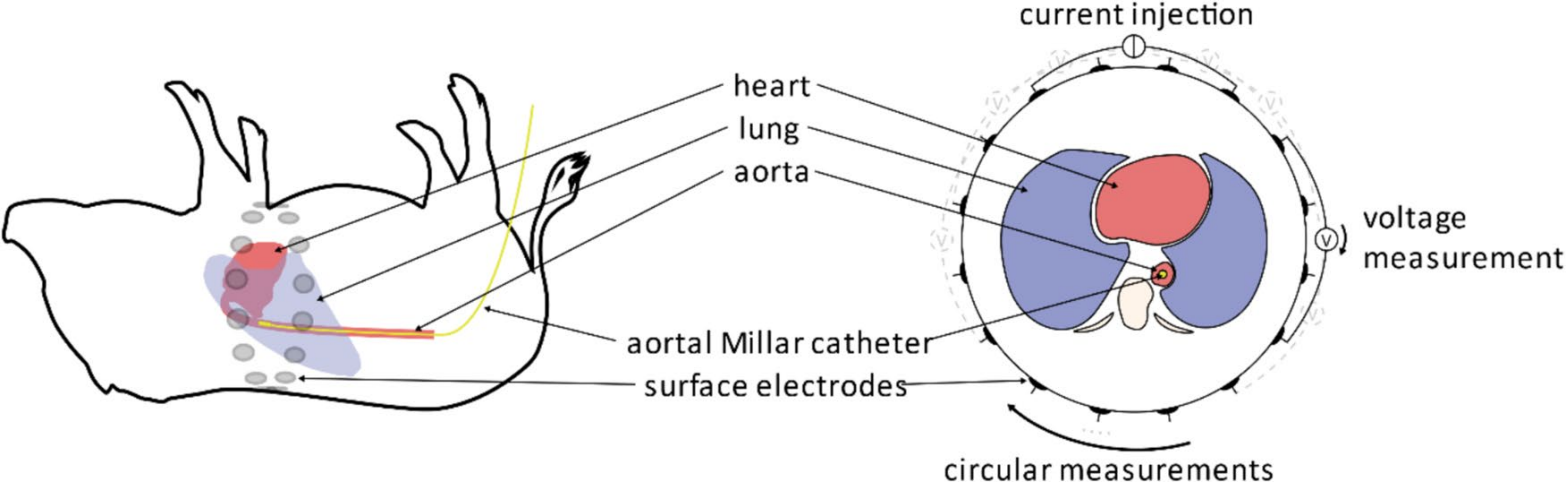
Schematic drawing of a thorax with an EIT belt placed directly over the heart (light red) and the lungs presented in blue. The descending aorta is shown in red with the tip of the Millar catheter (yellow) exactly in the plane of the EIT belt. On the right a tomographic view of heart, lungs and catheter it the EIT belt plane is presented

All pigs were prepared, anaesthetized, and mechanically ventilated according to local standards. They received a 4 Charrière (Ch) 16 cm PiCCO® (Getinge AB, Gothenburg, Sweden) catheter or an arterial line of the same size via the right femoral artery, a 5 Ch high-fidelity pressure sensor catheter (Micro-Tip® SPR-350, Millar Instruments Inc., Houston, TX, USA) in the descending aorta via the left femoral artery and a central venous catheter in the right internal jugular vein for hemodynamic monitoring. A custom-made elastic EIT belt with 32 electrodes was placed around the animal’s chest at the level of the heart (Fig. 1). A contact lubricant was used to improve electrical conductivity between the skin and the electrodes.

A key factor in ML applications is the quality of the reference data, which in case of the aortic pressure is provided by the catheter-based signals. The traditional fluid filled catheter usually provides stable measurements over time but tends to show fluid resonance effects [22, 24].

### Protocol

The thromboxane A2 (TXA) analogue U46619 (Enzo Life Sciences Science GmbH, Lörrach, Germany) was administered at doses of up to 0.15 µg kg^−1^ min^−1^ to induce experimental pulmonary hypertension by pulmonary vasoconstriction [14]. Temporary hypoxic vasoconstriction was also induced by reducing FiO2 to levels between 0.15 and 0.10 using Nitrogen (ALPHAGAZ™, Air Liquide Deutschland, Düsseldorf, Germany) [5], [17]. Lastly, a blood volume of 25 ml kg^−1^ of whole blood was withdrawn from each animal. The initial state (animals anesthetized, prior to intervention) served as control; therefore, no randomization was needed. Thereby potential confounders such as the order of treatments and measurements could not be completely excluded. A blinding of the investigator was not performed.

### Data acquisition and pre-processing

Physiological data were acquired at a sampling rate of 1 kHz using bridge transducer amplifiers in combination with dedicated hard- and software PowerLab 16/35, and LabChart 8 (both ADInstruments, Dunedin, New Zealand). The Millar catheter is known to present pressure curves without artifacts but to exhibit a drift over time, deviating considerably from the initial calibration. Therefore, the high-resolution pressure curves of Millar catheter needed to be recalibrated using the values of the fluid filled catheter. Systolic arterial pressure (SAP) was determined as the local maximum of the pressure curve within one heartbeat. Mean arterial pressure (MAP) was calculated as the arithmetic mean of pressures between two local minima, defined as the diastolic arterial pressure (DAP). Sequences containing premature heart beats were excluded from further analyses.^1^ EIT data were recorded using the Pioneer Set (EIT branch, Sentec AG, Landquart, Switzerland) connected to a custom-made 32-electrode EIT belt for pigs. EIT frames were sampled at a a rate of 47.68 Hz with each frame consisting of 1024 voltage values. The data presented here are not publicly available due to copyright issues, but can be made available on request.

**Fig. 2:**
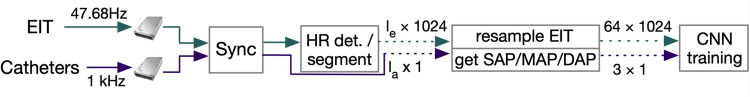
Schematic representation of the preprocessing steps applied prior to CNN training. EIT signals were recorded using the Pioneer Set at a sampling rate of 47.68 Hz, while blood pressures were recorded at 1 kHz using a PowerLab system. After synchronizing the physiological data with the EIT signals, heart rate was detected exclusively from the EIT signal. Based on this information both, the EIT signal and the physiological data was segmented to match the duration of a single heartbeat, aligning the corresponding systoclic, mean and diastolic arterial pressures. Thereafter these data were resampled and normalized in a block-wise manner. All heartbeats, regardless of their original duration, were reshaped to a uniform size of 64 × 1024, filling additional frames with zeros to ensure consistent sample length. Finally, machine learning training was conducted using such prepared data

**Fig. 3:**
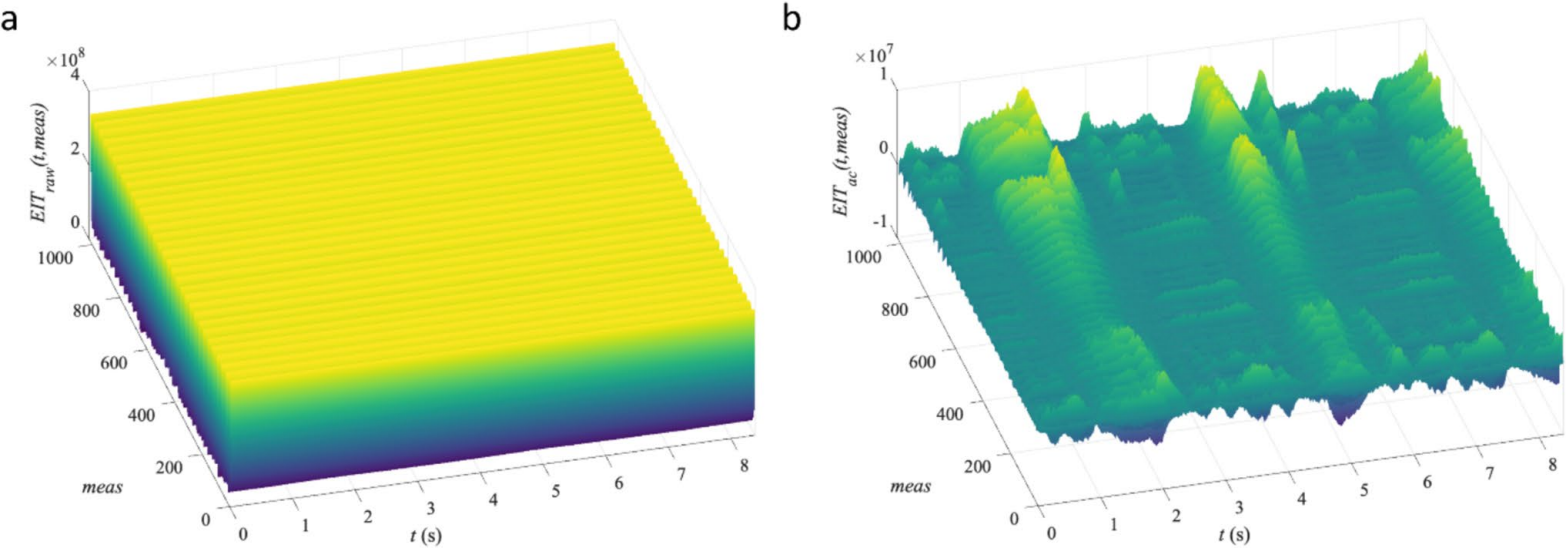
**a** depicts the real part of a representative RAW EIT dataset at 47.68 Hz over 8.4 s for a 32-electrode belt and the resulting 1024 measurements per frame. The same dataset after DC removal is shown in case **b** where ~ 2.5 ventilation cycles are most prominent. Additionally, the superimposed cardiac activity remains visible in a subset of the 1024 channels

The preprocessing steps prior to the CNN training are presented in Fig. 2. The raw EIT signal before and after AC removal is displayed in Fig. 3.

Due to different sampling rates and system clock states, synchronization between the EIT measurements and LabChart data had to be ensured by external means. Therefore, one of the PowerLab channels was dedicated to a timing reference signal from the EIT device. Every frame was linked to an additional manual trigger to mark the start and end of each measuring sequence. With an EIT frame sampling rate of 47.68 Hz and 1024 samples per frame, the alignment accuracy of the rising edge of the synchronization pulses was given by the 1 kHz resolution of the LabChart timing.

EIT data and reference signals were partitioned into segments each representing one heartbeat. Segments were identified from a spectral analysis of EIT data. Specifically, a Fourier-based Synchrosqueezing transformation was applied to obtain strongly localized frequencies and their harmonics corresponding to heartbeat and respiration [19]. The resulting spectrogram was iteratively parsed to track the instantaneous heartrate across time [13]. Since the length of these segments varied across time, data were resampled to ensure a constant size of the ML model input. The average segment length was 33 EIT frames, with very few segments exceeding 64 frames. Therefore, segments were re-sampled to a constant number of 64 EIT frames, and segments longer than 64 frames were excluded. This preprocessing is related in a size of the ML network input of 64 × 1024. To improve the model's reliability for generalization and prediction, a block-wise z-score normalization of the EIT data was performed. This process involved calculating the mean and standard deviation of each block, subtracting the mean, and dividing by the standard deviation. The aortic pressure waveform segments were used to extract the SAP, MAP and DAP values, which were then normalized to a range between 0 and 1 and stored as a 3 × 1 shaped vector.

### Machine learning model and model architecture

**Fig. 4:**
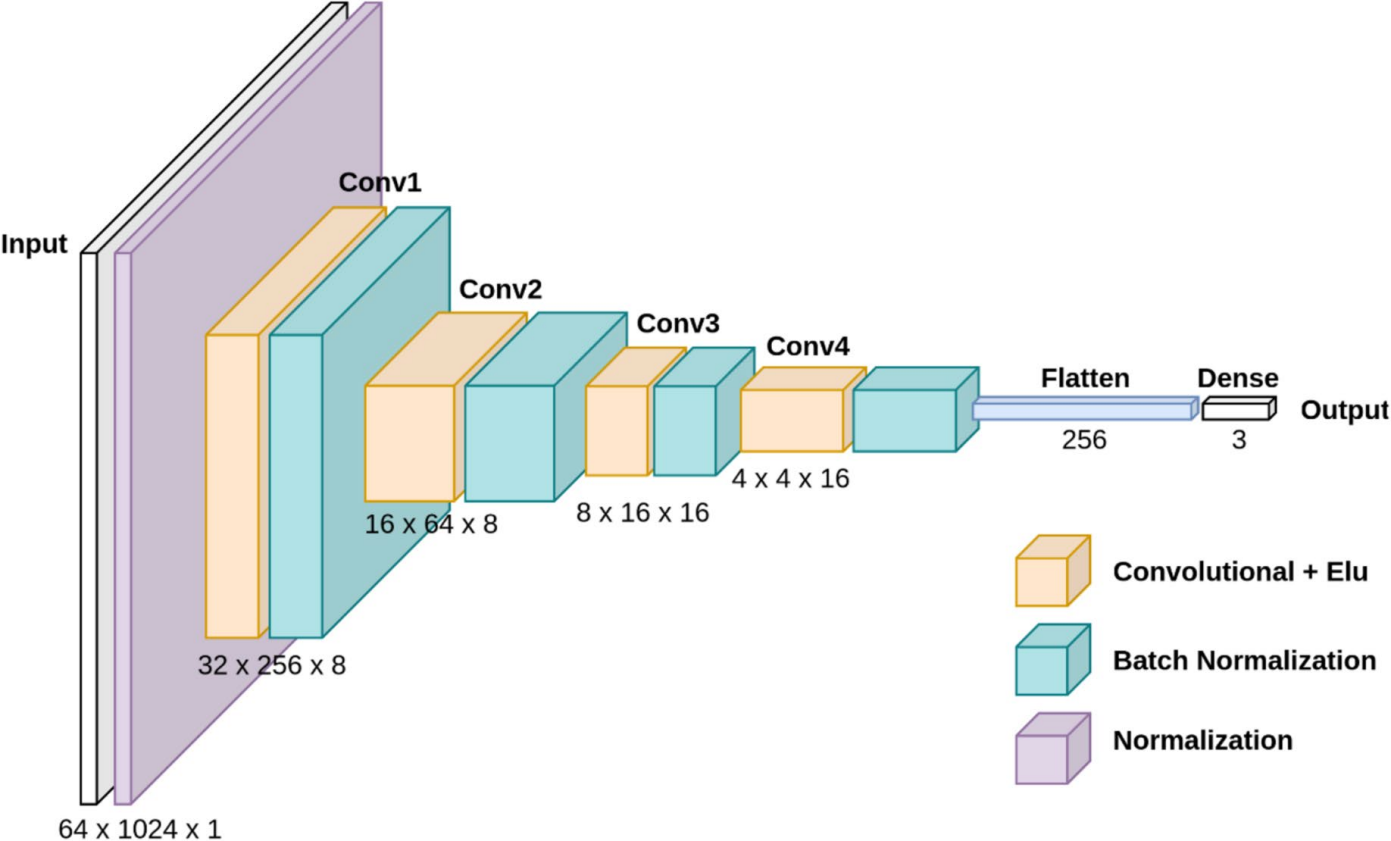
Network structure for predicting SAP, MAP and DAP from EIT measurements

The applied ML model for predicting SAP, MAP and DAP was a supervised trained convolutional neural network (CNN). The funnel-shaped dimension reduction of the CNNs had to extract and learn the prominent features from the EIT data (Fig. 4). Each convolution layer was followed by a batch normalization layer. The model's output consisted of a flattened layer that reduced the output of the last CNN to a one-dimensional vector, followed by a dense output layer. The kernel size of the convolutional layers was kept constant at 12 and the mean squared error (MSE) loss function was used during training. The filter size of the layers was decreased in the steps (8, 4, 2). In the dense output layer, the scalar values for SAP, MAP, and DAP were determined from these extracted EIT data features. The entire model had 39 996 parameters 37 851 of which were trainable.

**Fig. 5:**
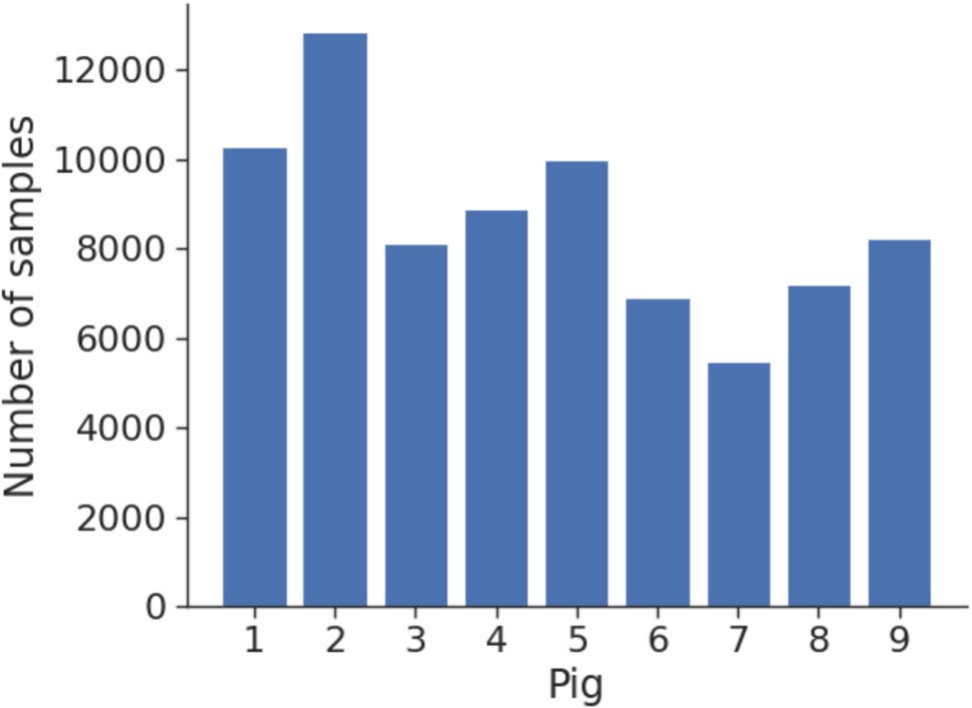
Histogram showing each animal’s contribution to the dataset of the 75 298 heartbeats used in this analysis

The network parameters needed to be adjusted such that an accurate mapping of EIT data to the corresponding pressure values was found. This process is termed training and was followed by a validation and a test phase. A well generalizing model should be able to predict blood pressure from EIT measurements of pigs not included in the training phase. Data from all nine pigs were of sufficient quality to deliver a total of 75 298 heartbeat samples. Each data sample contained the SAP, MAP and DAP values with the corresponding EIT measurements of one heartbeat. The amount of data samples per pig were not evenly distributed (Fig. 5). The dataset was divided into three parts: training, validation, and test sets. Training and validation sets comprised all samples from eight pigs, with a split of 90% for training and 10% for validation while and the dataset of the respective nineth pig was used for testing. The model was trained using the training dataset with randomly shuffled segments. Then, the trained model predicted the validation data, which consisted of unknown samples from known pigs, to evaluate overfitting and to verify successful training, where success was defined as a convergence of the loss function. In the final stage, the completely unknown test data from the nineth pig was predicted. If these predictions were satisfactory, the model's generalization was successful. In this investigation a k-fold cross-validation was performed by using each of the nine pigs as a test dataset and training the model on the data from the remaining eight pigs. If all models predicted the test pigs’ values with appropriate accuracy, it’s considered to have good generalization. To enhance the concordance between reference and estimated blood pressures, we conducted a hyperparameter tuning focusing on optimizing the variables known to influence the generalization of a trained model. As MAP is the most crucial parameter in the clinical setting for guiding therapeutic decisions, using ICC calculations we selected the model during the hyperparameter tuning which provided the best results for this parameter.

The training was run for 100 epochs with a batch size of 100 and an early stopping with a patience of five. After the training procedure, the model was validated with the validation data set to check whether it was able to determine the values of the respective blood pressure for unknown EIT measurements from known pigs. Afterwards, the model was tested with the test set. Finally, a hyperparameter tuning procedure was conducted for the purpose of implementing a systematic parameter adjustment with the objective of enhancing the accuracy of the model. Training was performed on a server at the University of Rostock equipped with eight NVIDIA A100-SXM4-80 GB (Nvidia Corporation, Santa Clara, California, USA) graphics cards.

### Statistics

Data were analyzed using SPSS Statistics 27 (IBM Corp., Armonk, New York, USA). Reference blood pressures for SAP, MAP and DAP were described by calculating the mean, standard deviation (SD) and variance. The Intraclass Correlation Coefficient (3,1) with absolute agreement (ICC) was computed to address discrepancies in the mean values, providing insight into the comparability of both analysis techniques [27]. ICC results were categorized as suggested by Koo et al. with values less than 0.5 as poor, between 0.5 and 0.75 as moderate, between 0.75 and 0.9 as good and greater than 0.90 as excellent reliability [10]. In a Scatterplot model and measured blood pressures are presented with a color coded heatmap showing the overlapping density displayed in 5 relative zones of frequencies from black to yellow. The potential harmfulness of blood pressures estimated non-invasively by EIT using ML was categorized with an error grid in this scatterplot into five risk zones according to Saugel et al. and classified as zone A with no risk for the patient, zone B low risk, zone C moderate risk, zone D significant risk and zone E dangerous risk for the patient [22]. Furthermore, agreement was examined utilizing Bland–Altman plots for repeated measurements with outcomes reported as mean bias and the limits of agreement (LoA) [4]. Plots were created using the Matplotlib of Python.

## Results

**Fig. 6:**
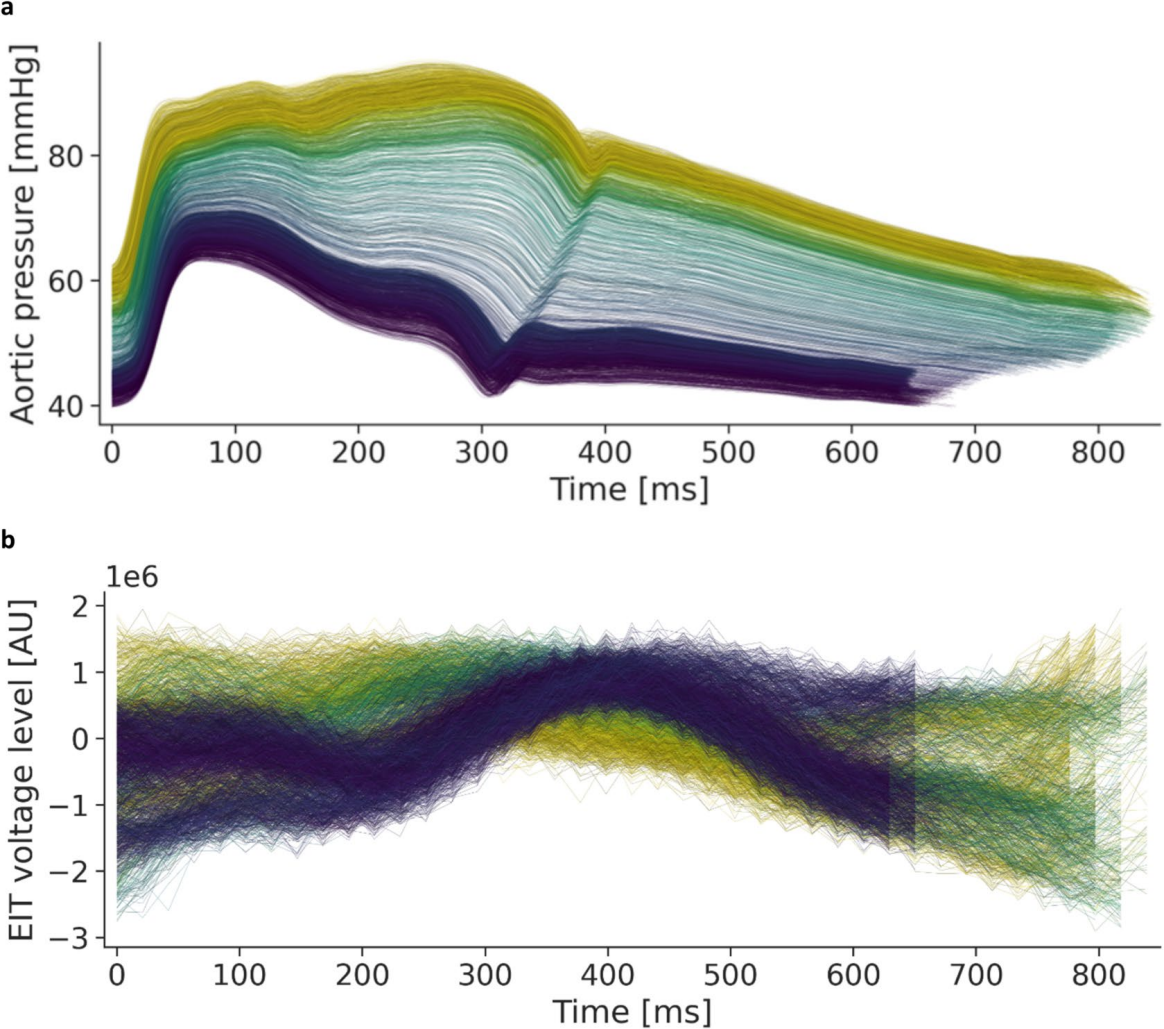
Panel a: Aortic pressure curves for each heartbeat of one animal with an individual color gradient illustrating the shift from higher (yellow) to lower blood pressures (blue) defined by the SAP. Panel b: global EIT signal corresponding to the aortic pressures using the same colors as above

In nine animals, 75 298 heartbeats with simultaneous arterial pressures and the concomitant EIT signal were eligible for training and testing of the ML algorithm (Fig. 5). Reference SAP values ranged from 22 to 179 mmHg, with a mean of 104 mmHg (SD 19.7) and a variance of 387. Reference MAP values ranged from 13 to 146 mmHg, with a mean of 90.2 mmHg (SD 18.7) and a variance of 348. Reference DAP values ranged from 5 to 119 mmHg, with a mean of 74 mmHg (SD 17.2) and a variance of 294. Reference pressure curves of one animal are depicted in Fig. 6a while 6b shows the corresponding EIT signal used by the ML-trained algorithm to predict the pressure categories SAP, MAP, and DAP for the same animal. The EIT-based predictions resulted in an ICC for SAP of 0.530, for MAP of 0.563, and for DAP of 0.521. The Correlation Coefficients for the chosen model were 0.435 for SAP, 0.476 for MAP and 0.436 for DPA.

**Fig. 7:**
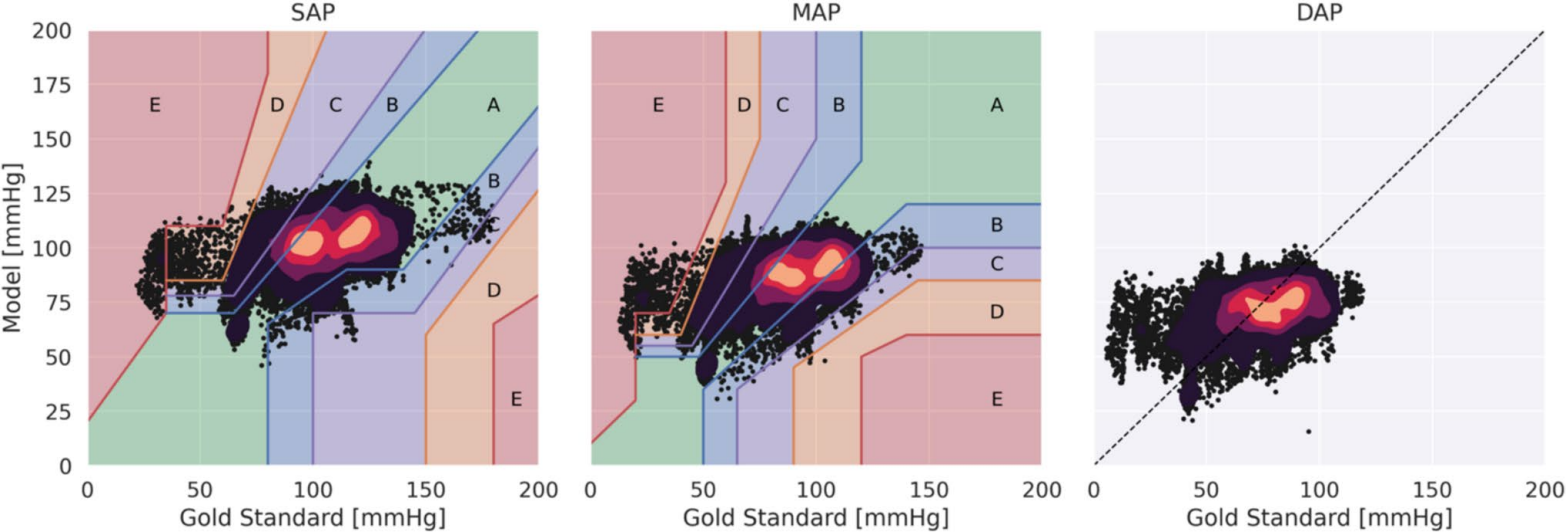
Scatter plots of SAP, MAP and DAP for all heratbeats of the individual test pigs of the k-fold cross-validation. Plots compare pressure estimates from the Machine Learning model with measurements obtained via a compensated microtip catheter. Background color map represents the risk gradient for non-invasively estimated blood pressures as suggested by Saugel et al., where zone A represents no risk for the patient, zone B a low risk, zone C a moderate risk, zone D a significant risk, and zone E a dangerous risk [22]. For the DAP such risk gradients were not available. Overlapping data points in the scatter plots are presented by a heatmap in 4 steps from purple to orange in 20% steps each

**Fig. 8:**
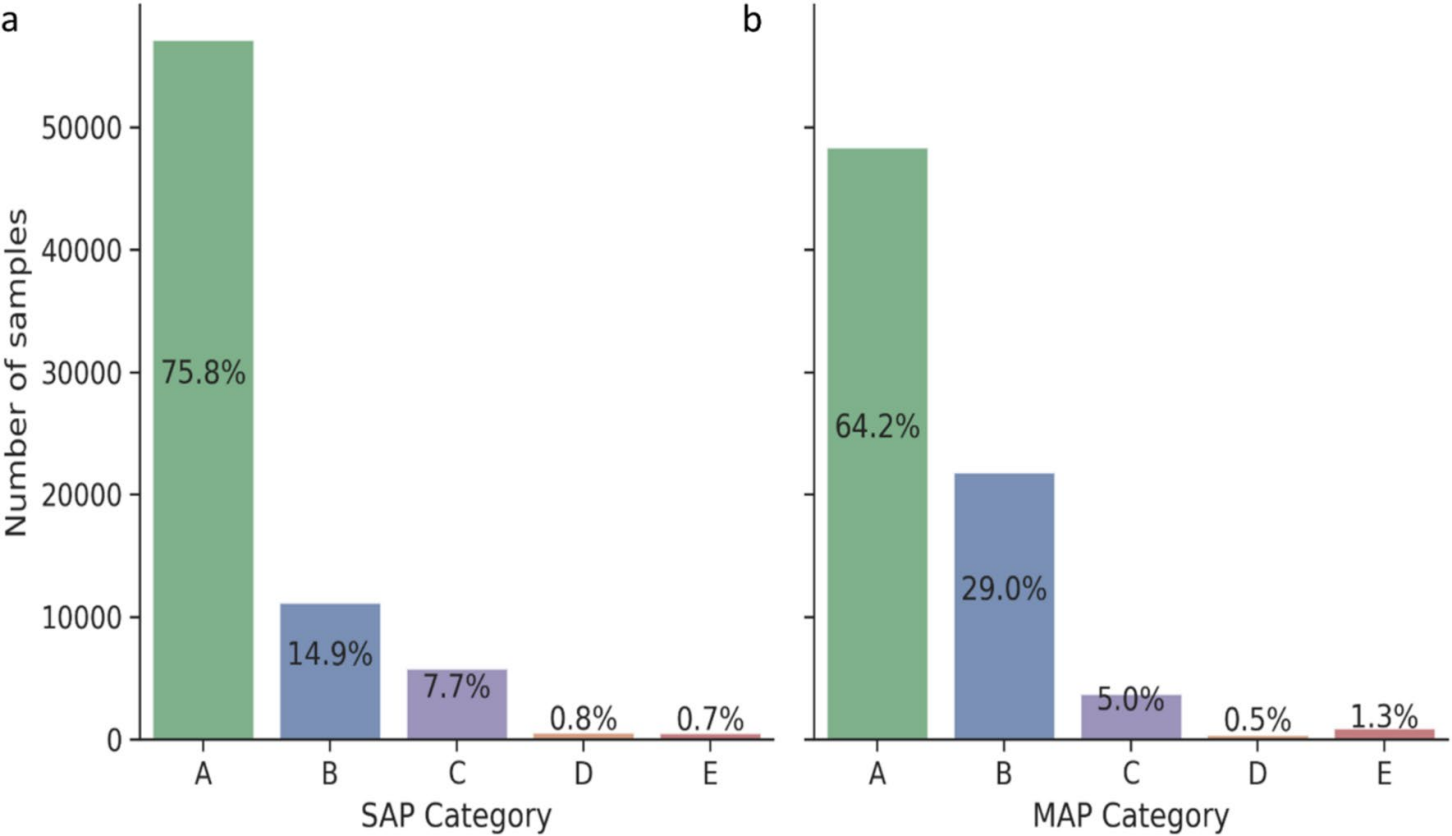
Panels a and b: histograms of the risk zones for SAP and MAP, as defined by Saugel et al., comparing non-invasive pressure estimates with blood pressures measured by the gold standard (for risk categories see previous legends) [22]. 75.8% of SAP estimates were within category A and 64.2% of MAP

**Fig. 9:**
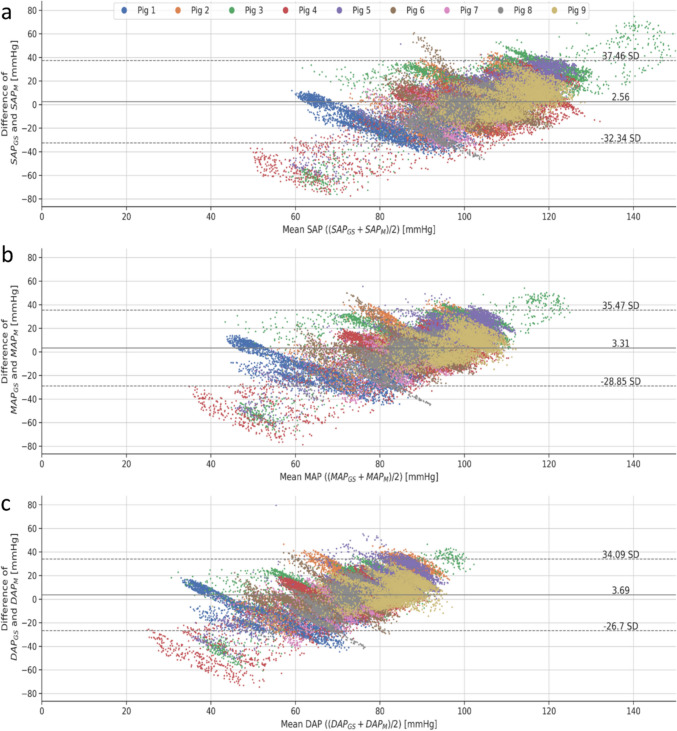
Bland–Altman plots for SAP (**a**), MAP (**b**) and DAP (**c**) with data from each one of the animals depicted in an individual color. The continuous horizontal line represents the mean difference, while the upper and lower dashed lines delineate the limits of agreement corresponding to ± 1.96 standard deviations of the mean difference between gold standard (GS) and model (M). The plots reveal a low mean bias between ML-based blood pressure estimations and invasively measured blood pressures. The mean bias for SAP was 2.56 mmHg (LoA: −32.34 to 37.46 mmHg), for MAP 3.31 mmHg (LoA: −28.85 to 35.47 mmHg) and for DAP 3.69 mmHg (LoA: −26.70 to 34.09 mmHg), respectively

**Fig. 10:**
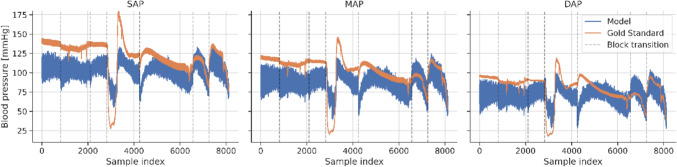
Trending plot of the EIT-based ML-predicted aortic SAP, MAP and DAP throughout an entire experiment for a single animal, demonstrating the method's ability to capture trends over time. Each measuring segment is separated by a block transition

**Fig. 11:**
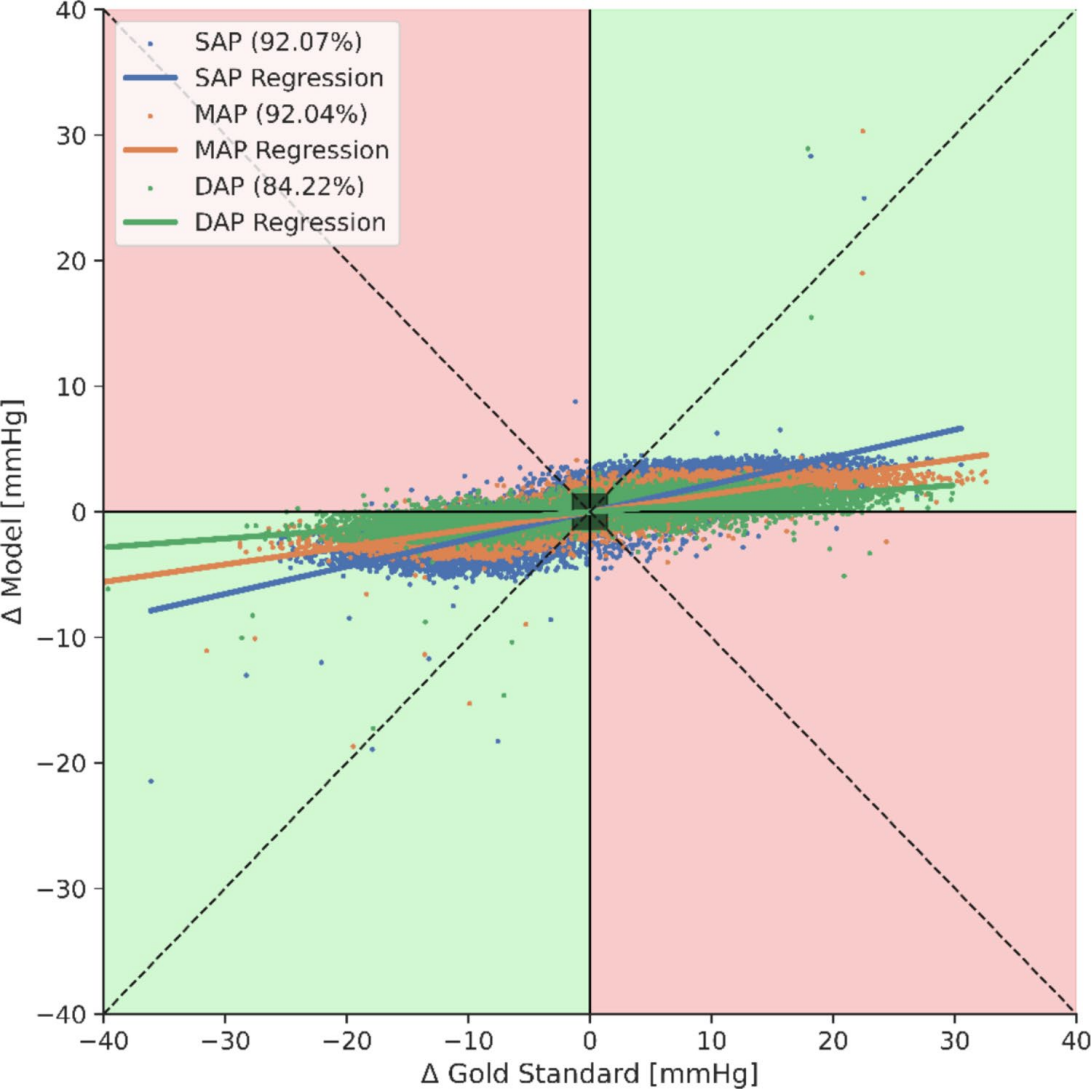
4-Quadrant Plot for SAP, MAP and DAP for all heartbeats of the animal shown in Fig. 10 representing the relative changes of measured and estimated blood pressures. The exclusion zone represents 10% of the maximal systolic change. The concordance rate was 92,1% for SAP, 92,0% for MAP and 84,2% for DAP

Figure 7 depicts the SAP, MAP and DAP estimated from EIT measurements versus the respective gold standard invasive measurements for all analyzed heartbeats. A color code from black to yellow shows overlapping data points with increasing density. For SAP and MAP, these scatterplots were plotted within a previously published color map to categorize the quality of the predictions from A to E in 5 decreasing levels of risk [22]. For the selected ML model and all animals SAP and MAP were classified as grade A in 75.8% of the cases and 64.2%, respectively (Fig. 8). Analysis according to Bland–Altman is presented in Fig. 9. The model’s trending ability is shown in a representative animal as a chronological blood pressure profile for the hole experiment comparing measured and estimated blood pressures (Fig. 10). The 4-quadrant plot resulted in a concordance rate of 92% for SAP, 92% for MAP and 84% for DAP (Fig. 11).

## Discussion

Our results represent the first proof of concept how a ML strategy can estimate aortic blood pressure from thoracic EIT-voltage raw data. To the best of our knowledge, this is the first study utilizing thoracic voltages from a thoracic EIT application successfully to directly compute absolute blood pressures and not only relative pressure curve shapes [25]. The ML-derived blood pressures calculated from measured thoracic EIT-voltages were compared with the aortic reference pressures. For SAP 90,7% of the analyzed heartbeats showed a good concordance meaning that potential differences in blood pressures would not lead to another treatment (risk zone A and B) [22]. This was also the case for MAP in 93,2% of the analyzed heartbeats [22].

The significant proportion of measurements falling into zone A and B highlights the potential of the method employed in this study for assessing blood pressures in anesthetized pigs. Furthermore, the very few pressure estimates in zones D and E can be considered outliers underscoring the potential safety of this approach [22]. With a mean bias below 5 mmHg the average comparability for SAP, MAP and DAP was very good although the limits of agreement were rather wide. The Bland–Altman plot indicates that the model tended to underestimate higher pressure values and overestimated lower ones. Such inaccurate predictions could result in harmful clinical decisions. For instance, if the model estimated that a patient’s blood pressure is high when in fact it is low, the clinician might administer antihypertensive medications instead of the catecholamines the patient truly needs to maintain sufficient blood pressure. Such an error in treatment choice could have serious consequences for patient safety. Since, our results show a trending ability of our method when the chronological history of the raw data is taken into account, it is likely that occasional errors in blood pressure might be found. The model successfully detected periods of rising and falling blood pressure in response to hemodynamic changes, despite phases with stable offsets. Such trending will at least in part mitigate the above shortcomings of the absolute pressure estimations and will prevent clinicians from misinterpreting the real hemodynamic situation of the patient.

Current EIT-systems inherently lack the ability to measure absolute impedances [3]. Thus, the ML-based algorithm can only work with relative changes in thoracic impedance during the course of one heartbeat. Yet, there is clear evidence that such characteristic impedance changes are induced by different aortic blood pressures [1]. These relative changes are due to shifts in blood volume along the cardiac cycles and a propagation of blood into the aorta [1]. This pulse wave propagation is blood pressure depended with higher pressures leading to a faster pulse wave propagation and lower pressures to a slower propagation. Thereby, the thoracic impedance changes, representing a cyclic redistribution of a highly conductive volume of fluid—blood—is inherently tied to blood pressure. Although it is not obvious which characteristics of the EIT measurements are learned by the ML model, results show that there is a physiological link between EIT measurements and aortic blood pressure.

Our approach of using raw EIT voltage data eliminates the need for reconstructing EIT images and subsequent analysis of such images [25]. By bypassing these conventional steps this approach may overcome the inevitable loss of information during the reconstruction, streamline the process and reduce the computational load, which are notable advancements in the use of EIT technology for real-time monitoring.

In our experimental study, we sought to extend the capabilities of ML by exploring its potential to predict arterial blood pressures directly from EIT voltage data instead of analyzing EIT images. This study establishes the connection between a hemodynamic parameter and voltages measured non-invasively at the body surface, thereby linking physiological phenomena with thoracic conductivity changes. Although our results from an experimental study in pigs underscore the ML’s potential to estimate arterial pressures from EIT data, we acknowledge the inherent and even bigger challenges associated with implementing ML in clinical practice such as data paucity and variability, model interpretability, and generalizability across diverse patient populations [28].

To apply an entirely new approach of ML to the estimation of blood pressures from voltages of thoracic EIT we decided to use a CNN. It was chosen because this type of network architecture has significantly fewer parameters than a fully connected deep neural network (DNN) with the same input and output dimensions. The funnel-shaped dimension reduction of the CNNs extract and learn the prominent features from the EIT data. To this approach a block wise z-score normalization layer was added with the advantage of allowing data to be normalized within smaller, more manageable blocks or segments, rather than normalizing the entire dataset at once. This normalization is meaningful because the targeted information is not contained in the absolute amplitudes of EIT data. Moreover, it improves the learning process particularly. This normalization approach is useful when dealing with data that exhibited significant variations or fluctuations, such as in this pig study dataset [8].

Beyond arterial pressures, ML could also be employed to estimate other hemodynamic parameters non-invasively such as cardiac output [20], stroke volume variation [12], pulmonary artery pressure [21], and extra alveolar lung water [26] for which EIT is known to present some composite signal information. By training ML models on large datasets containing EIT signals and corresponding hemodynamic measurements, it may be possible to develop accurate models capable of determining various cardiovascular parameters.

Our investigation has several limitations: Over all, the ML model does not yet perform at a level of accuracy and reliability to meet clinical safety demands. It is so far not interchangeable with reference measurements of the blood pressure. This has some specific reasons: First of all, the sample size with only nine animals was very small. The use of uniformly young landrace pigs does not account for variations in age, gender, or comorbidities, further constraining the generalizability of our findings. In the context of the hyperparameter tuning, the training of the ML model was based on eight out of nine pigs, only. The nineth pig was used for testing. For the cross-validation, this procedure was repeated such that every pig was chosen once for testing. As a result, each individual animal has been tested by a slightly different model. Therefore, the correlation between estimated and measured blood pressures may improve if the training dataset included a larger and more diverse set of individuals. While the number of animals in this study was low, over 70,000 individual heartbeats over a wide range of blood pressures were included (compare Fig. 5). Since the number of heartbeats stemming from each pig was not equal our recordings have to be considered an unbalanced dataset.

It was our aim to measure blood pressures with the best equipment available at the level where the EIT belt plane crosses the descending aorta. While the exact location could be verified by radiographic means, we faced an offset in the pressures measured by the Millar catheter that could not be eliminated by (repeated) two-point calibrations in all animals. Therefore, we had to correct the Millar measurements by the ones with the fluid filled external disposable pressure sensor. This compensation was adjusted for the DAP and applied to all selected heartbeats.

Nevertheless, most of the analyzed heartbeats included in our experiments fall within typical normotensive conditions. As a result, the majority of data points cluster around a SAP of approximately 110 mmHg and a MAP of about 90 mmHg. This uneven distribution may unintentionally aid the algorithm, since predicting a normotensive blood pressure is statistically more likely to be correct. Future studies should not only investigate this approach under hypotensive conditions, but also expand the testing to hypertensive states. Additionally, data collection should target predefined equally distributed pressure ranges to achieve a more uniform distribution across the entire spectrum of possible blood pressures, thereby strengthening the quality of the results.

## Conclusion

A machine learning model utilizing thoracic voltages from EIT might predict in part aortic blood pressures in experimental pigs whose data were unknown to the algorithm. Our study highlights the transformative potential of ML in EIT-based hemodynamic monitoring.

## Data Availability

The data presented in this study are available on reasonable request from the corresponding author. The data are not publicly available due to copyright issues.

## Footnotes

1 Usually, images illustrating dynamic lung and heart activities are constructed from EIT measurements. Instead, the measured voltages between electrodes (raw data) are directly processed without image reconstruction.
